# Effect of data conserving respiratory motion compensation on left ventricular functional parameters assessed in gated myocardial perfusion SPECT

**DOI:** 10.1186/s40658-021-00355-w

**Published:** 2021-01-21

**Authors:** Matti J Kortelainen, Tuomas M Koivumäki, Marko J Vauhkonen, Mikko A Hakulinen

**Affiliations:** 1 Department of Applied Physics, University of Eastern Finland, POB 1627, Kuopio, FI-70211 Finland; 2 Diagnostic Imaging Center, Kuopio University Hospital, Kuopio, Finland; 3 Department of Medical Physics, Central Finland Central Hospital, Jyväskylä, Finland

**Keywords:** SPECT, Myocardial perfusion imaging, Respiratory motion, Motion compensation, Phase analysis

## Abstract

**Background:**

Respiratory motion compromises image quality in myocardial perfusion (MP) single-photon emission computed tomography (SPECT) imaging and may affect analysis of left ventricular (LV) functional parameters, including phase analysis-quantified mechanical dyssynchrony parameters. In this paper, we investigate the performance of two algorithms, respiratory blur modeling (RBM) and joint motion-compensated (JMC) ordered-subsets expectation maximization (OSEM), and the effects of motion compensation on cardiac-gated MP-SPECT studies.

**Methods:**

Image acquisitions were carried out with a dual-detector SPECT/CT system in list-mode format. A cardiac phantom was imaged as stationary and under respiratory motion. The images were reconstructed with OSEM, RBM-OSEM, and JMC-OSEM algorithms, and compared in terms of mean squared error (MSE). Subsequently, MP-SPECT data of 19 patients were binned into dual-gated (respiratory and cardiac gating) projection images. The images of the patients were analyzed with Quantitative Gated SPECT (QGS) 2012 program (Cedars-Sinai Medical Center, USA). The parameters of interest were LV volumes, ejection fraction, wall motion, wall thickening, phase analysis, and perfusion parameters.

**Results:**

In phantom experiment, compared to the stationary OSEM reconstruction, the MSE values for OSEM, RBM-OSEM, and JMC-OSEM were 8.5406·10^−5^,2.7190·10^−5^, and 2.0795·10^−5^, respectively. In the analysis of LV function, use of JMC had a small but statistically significant (*p* < 0.05) effect on several parameters: it increased LV volumes and standard deviation of phase angle histogram, and it decreased ejection fraction, global wall motion, and lateral, septal, and apical perfusion.

**Conclusions:**

Compared to standard OSEM algorithm, RBM-OSEM and JMC-OSEM both improve image quality under motion. Motion compensation has a minor effect on LV functional parameters.

**Supplementary Information:**

The online version contains supplementary material available at (10.1186/s40658-021-00355-w).

## Background

Myocardial perfusion (MP) single-photon emission computed tomography (SPECT) examination is often performed in the diagnosis of coronary artery disease [1]. Cardiac gating of MP-SPECT examination further enables objective assessment of left ventricular (LV) functional parameters [2], which provide additional information in, for example, diagnosis of myocardial infarction [3], identification of multiple-vessel disease [4], prediction of mortality [5], and identification of patients with risk for LV remodeling [6]. In addition, evaluation of LV mechanical dyssynchrony with phase analysis [7] may help to interpret whether the patient would benefit from cardiac resynchronization therapy [8, 9].

During the MP-SPECT examination, the heart undergoes quasiperiodic motion due to respiration; this movement can even exceed 20 mm [10]. As a result, the reconstructed SPECT images appear increasingly “blurred” if the motion is not accounted for in the reconstruction. Fortunately, several motion compensation methods have been introduced over the years [11–15]; however, as far as we know, there are only few papers where the effect of respiratory motion compensation on analysis of LV function has been investigated [16, 17]. In addition, only one of these studies investigated the effect of motion compensation on phase analysis results [17]. What is more, the motion compensation method applied in reference [17] was essentially a respiratory gating method, which means that the resulting images had amplified noise levels compared to the concurrent clinical standards.

The aim of this study is to investigate the effect of respiratory motion compensation on results obtained from cardiac-gated MP-SPECT examinations. First, a cardiac phantom is imaged to validate two promising motion compensation strategies. Subsequently, these motion compensation methods are applied on clinical cardiac-gated MP-SPECT data. Finally, the clinical images reconstructed with the better motion compensation method are analyzed in terms of LV functional parameters and compared to standard clinical reconstructions. Specifically, the effect of motion compensation on phase analysis parameters is investigated.

## Methods

### Data acquisition and preprocessing

All image acquisition procedures were carried out on a dual-detector SPECT/CT system (Precedence; Royal Philips N.V., Netherlands) at Kuopio University Hospital. All SPECT data were acquired in list-mode format and processed in MATLAB environment (MATLAB R2015b; The MathWorks, Inc., MA, USA).

#### Phantom experiment

A custom-designed phantom, which consists of a cardiac phantom and a “torso” phantom, was utilized in this part of the study [18]. The cardiac phantom was filled with 121 MBq of Tc-99m and placed inside the “torso” phantom. The “torso” phantom was then placed on a plastic plate on top of the patient table of the SPECT/CT system and attached to a respiratory phantom (AZ-733V; Anzai Medical Co.,Ltd., Japan). The respiratory phantom provided a sinusoidal motion pattern with 10 RPM frequency and 20 mm peak-to-peak amplitude. The motion was directed parallel to the rotation axis of the SPECT/CT system.

Before SPECT imaging, the phantom was scanned with CT. The following standard clinical settings were used: helical scan mode, tube voltage of 140 kV, tube current-exposure time product of 20 mAs, matrix size of 512 ×512, slice thickness of 5 mm, and pixel size of 1.171875 mm. The phantom was stationary during the CT scan.

The phantom was imaged twice: first with respiratory motion and then as stationary (reference scan). Standard clinical myocardial perfusion imaging acquisition protocol of Kuopio University Hospital was used. This includes 90^∘^ detector configuration, noncircular detector orbit with 64 steps from right anterior oblique to left posterior oblique angle, low-energy high-resolution collimators, energy window of 140 keV ± 10%, and acquisition time of 30 s per step.

To acquire respiratory signal for gating, the method of Sakaguchi et al. was applied [19]. Briefly, the list-mode data were binned into 50-ms frames from which the center of activity (COA) in the axial direction (*z*-axis) was computed. Since it was known that the phantom motion is sinusoidal, a sine wave was fit to the COA data using a Gauss-Newton scheme. This sine wave thus represented the phantom’s axial position through the entire image acquisition.

In order to induce a more realistic respiratory pattern for the phantom study, distribution of acquisition time among the 64 projection angles and 10 equal-sized respiratory amplitude windows was assessed from one clinical patient data. This distribution was used to scale the phantom’s acquisition time correspondingly when the list-mode data was binned into respiratory-gated projection images. Total acquisition time per projection angle applied in the binning was 4.542 s, the matrix size was 128 ×128, and the pixel size was 4.664 mm. Since the stationary scan was acquired after the moving scan, to account for decay, the acquisition time per projection angle applied in the binning was increased to 4.812 s.

#### Patient studies

The study population consisted of 19 patients (8 female) referred to 1-day clinical stress/rest MP-SPECT examinations. Their characteristics (mean ± standard deviation) were as follows: age, 69 ± 11 years; weight, 76 ± 13 kg; height, 169 ± 12 cm; and body mass index, 26.4 ± 2.9 kg/m^2^. The patients received 300 MBq of Tc-99m tetrofosmin prior to the stress imaging and 706 ± 11 MBq prior to the rest imaging 3 h later. For the purposes of this study, data were collected at rest only. Written informed consent was obtained from every participating patient, and the study was approved by the Research Ethics Committee of the Northern Savo Hospital District (Dno 90/2011; March 20, 2012).

Image acquisition was started 45 min after the rest injection. The patients’ electrocardiogram (ECG) was monitored with a cardiac trigger monitor (CTM 3000; Ivy Biomedical Systems, Inc., USA). The patients were imaged in a supine position at each step until 30 s of R-R intervals within a 40%-wide ECG gating acceptance window had been acquired. Otherwise, the image acquisition protocol was the same as in the phantom experiment.

ECG and thoracic electrical bioimpedance (EBI) of each patient were measured with a data acquisition system equipped with amplifiers (MP150, ECG100C, and EBI100C; BIOPAC Systems, Inc., USA). Since the data acquisition system had to be started earlier than the SPECT/CT system, there was a delay *Δt* between the recorded signals and the list-mode data. To synchronize the signals with the list-mode data, the R-triggers from the cardiac trigger monitor were transferred simultaneously to the SPECT/CT system and the data acquisition system via an in-house built pulse generator. The delay *Δt* was found by maximizing the cross-correlation of these two resulting R-trigger sequences, after which the ECG and EBI signals could be shifted in time to match the list-mode data.

For cardiac gating, R waves were detected from the ECG signal using Pan-Tompkins method [20] and the R-R intervals were divided to 8 cardiac bins using forward gating approach with fixed bin length [21]. The EBI signal was low-pass filtered after which non-physiological trends were removed with a moving average filter [17]. The filtered EBI signal was treated as the respiratory signal which was used to compute respiratory gating limits according to Dey et al. [22]. Seven equal-sized amplitude windows were used because for the majority of patients the measured respiratory motion magnitude was less than 14 mm, and a previous study suggested that respiratory blur of an individual respiratory window should be less than 2 mm [18]. Thus, ECG and EBI signals were used to bin list-mode data into a total of 3584 dual-gated projection images (8 cardiac bins ×7 respiratory windows × 64 projection angles) with a pixel size of 6.219 mm.

### Reconstructions

We present the reconstruction-related equations for the dual-gated data, noting that the phantom data are also dual-gated data—with a single cardiac bin.

#### Parameters

For the sake of comprehensibility, we first present all the parameters required in the following sections. Let *C*, *R*, and *L* denote the number of cardiac bins, respiratory windows, and projection angles, respectively. Let *M* and *N* denote the number of pixels in a projection image and number of voxels in the unknown/estimated image, respectively. Let $\tau _{c,r}^{\ell }$ denote the image acquisition time at cardiac bin *c*, respiratory window *r*, and projection angle *ℓ*. Let

$$\boldsymbol{g}_{c,r}^{\ell} \in \mathbb{R}^{M \times 1}, \ c = 1,\dots,C, r = 1,\dots,R, \ell = 1,\dots,L $$

denote the dual-gated projection image at cardiac bin *c*, respiratory window *r*, and projection angle *ℓ*. Let

$$\boldsymbol{f}_{c} \in \mathbb{R}^{N \times 1}, \ c = 1,\dots,C $$

denote the unknown, true image at cardiac bin *c* and let

$$\hat{\boldsymbol{f}}_{c} \in \mathbb{R}^{N \times 1}, \ c = 1,\dots,C $$

denote the estimated image at cardiac bin *c*. Let

$$\boldsymbol{K}_{c,r}^{\ell} \in \mathbb{R}^{M \times N}, \ c = 1,\dots,C, r = 1,\dots,R, \ell = 1,\dots,L $$

denote the observation matrix block at cardiac bin *c*, respiratory window *r*, and projection angle *ℓ*. Let ***g***_*c*,*r*_ denote a concatenated vector such that

$$\boldsymbol{g}_{c,r} = \left[[\boldsymbol{g}_{c,r}^{1}]^{T},[\boldsymbol{g}_{c,r}^{2}]^{T},\dots,[\boldsymbol{g}_{c,r}^{L}]^{T}\right]^{T} \in \mathbb{R}^{ML \times 1} $$

and let ***K***_*c*,*r*_ denote a concatenated matrix such that

$$\boldsymbol{K}_{c,r} = \left[[\boldsymbol{K}_{c,r}^{1}]^{T},[\boldsymbol{K}_{c,r}^{2}]^{T},\dots,[\boldsymbol{K}_{c,r}^{L}]^{T}\right]^{T} \in \mathbb{R}^{ML \times N}. $$

Let

$$\boldsymbol{P}^{\ell} \in \mathbb{R}^{M \times N}, \ \ell = 1,\dots,L $$

denote the forward projection matrix at projection angle *ℓ* and let

$$\boldsymbol{R}^{\ell} \in \mathbb{R}^{N \times N}, \ \ell = 1,\dots,L $$

denote a rotation matrix at projection angle *ℓ*. Finally, let $\boldsymbol {M}_{r'\rightarrow r} \in \mathbb {R}^{N \times N}$ denote a transformation matrix from a reference respiratory window *r*^′^ to respiratory window *r* and let $\boldsymbol {1} \in \mathbb {R}^{ML \times 1}$ denote a vector of ones.

#### Non-motion-compensated reconstruction

For reference, it is assumed that no respiratory motion occurs during the image acquisition and the data is not respiratory-gated. In this case, neglecting attenuation, scattered photons, and measurement errors, our imaging model is

1 $$  \sum_{r=1}^{R}\boldsymbol{g}_{c,r}^{\ell} = \left(\sum_{r=1}^{R} \boldsymbol{K}_{c,r}^{\ell} \right)\boldsymbol{f}_{c}  $$

where the observation matrix blocks are [23]

$$\boldsymbol{K}_{c,r}^{\ell} = \tau_{c,r}^{\ell}\boldsymbol{P}^{\ell}\boldsymbol{R}^{\ell}. $$

Images $\hat {\boldsymbol {f}}_{c}$ can now be reconstructed with maximum likelihood expectation maximization (MLEM) algorithm [24]

2 $$  \hat{\boldsymbol{f}}_{c}^{(\text{new})} = \frac{\hat{\boldsymbol{f}}_{c}^{(\text{old})}}{\left(\sum_{r=1}^{R}\boldsymbol{K}_{c,r}^{T}\right)\boldsymbol{1}}\left(\sum_{r=1}^{R}\boldsymbol{K}_{c,r}^{T}\right)\left(\frac{\sum_{r=1}^{R}\boldsymbol{g}_{c,r}}{\left(\sum_{r=1}^{R}\boldsymbol{K}_{c,r}\right)\hat{\boldsymbol{f}}_{c}^{(\text{old})}}\right),  $$

where superscripts (new) and (old) denote new and old estimates of $\hat {\boldsymbol {f}}_{c}$, respectively.

#### Motion-compensated reconstruction

**Respiratory blur modeling** The first motion compensation strategy that we apply has previously been used in positron emission tomography [25, 26]. It bases on an assumption that the data is motion-blurred (not respiratory-gated) and this is taken into account in the imaging model; therefore, we call it respiratory blur modeling (RBM). In practice, this means that imaging model (1) applies, and the observation matrix blocks are modified such that

3 $$  \boldsymbol{K}_{c,r}^{\ell} = \tau_{c,r}^{\ell}\boldsymbol{P}^{\ell}\boldsymbol{R}^{\ell}\boldsymbol{B}_{c}^{\ell},  $$

where $\boldsymbol {B}_{c}^{\ell }$ is a blurring matrix such that

$$\boldsymbol{B}_{c}^{\ell} = \sum_{r=1}^{R}\frac{\tau_{c,r}^{\ell}}{\sum_{r=1}^{R} \tau_{c,r}^{\ell}}\boldsymbol{M}_{r'\rightarrow r}. $$

The reconstruction is carried out with Eq. (2) using the modified observation matrix blocks (Eq. (3)).

**Joint motion-compensated reconstruction** The second motion compensation strategy that we apply was presented by Qi et al. [13] and is called joint motion-compensated (JMC) reconstruction. This method bases on a common gating assumption that the object to be imaged moves instantaneously from one position to another, but stays still while occupying a position. This means that the imaging model is

4 $$  \boldsymbol{g}_{c,r}^{\ell} = \boldsymbol{K}_{c,r}^{\ell}\boldsymbol{f}_{c},  $$

where the observation matrix blocks are

5 $$  \boldsymbol{K}_{c,r}^{\ell} = \tau_{c,r}^{\ell}\boldsymbol{P}^{\ell}\boldsymbol{R}^{\ell}\boldsymbol{M}_{r'\rightarrow r}.  $$

Derivation of JMC-MLEM algorithm follows the same path as that of the original MLEM algorithm [24]. That is, the solution is searched by formulating the likelihood function *p*(***X***_*c*_|***f***_*c*_) for the so-called complete data tensor ***X***_*c*_

$$p\left(\boldsymbol{X}_{c}|\boldsymbol{f}_{c}\right) = \prod_{r}\prod_{i}\prod_{j}\mathrm{e}^{\left(-\boldsymbol{K}_{c,r}(i,j)\boldsymbol{f}_{c}(j)\right)}\frac{\left(\boldsymbol{K}_{c,r}(i,j)\boldsymbol{f}_{c}(j)\right)^{\boldsymbol{X}_{c}\left(i,j,r\right)}}{\boldsymbol{X}_{c}\left(i,j,r\right)!}, $$

where ***X***_*c*_(*i*,*j*,*r*) is the number of photons emitted by image voxel *j* and collected at projection image pixel *i* at respiratory window *r* and cardiac bin *c*. Taking the conditional expectation of log-likelihood with respect to data vector ***g***_*c*,*r*_ and current estimate of ***f***_*c*_ (expectation step), and setting its derivatives with respect to the unknown values of ***f***_*c*_(*j*) to zero (maximization step), one has derived the JMC-MLEM algorithm. In matrix form, this becomes

6 $$  \hat{\boldsymbol{f}}_{c}^{(\text{new})} = \frac{\hat{\boldsymbol{f}}_{c}^{(\text{old})}}{\sum_{r=1}^{R}\boldsymbol{K}_{c,r}^{T}\boldsymbol{1}}\left(\sum_{r=1}^{R}\boldsymbol{K}_{c,r}^{T}\frac{\boldsymbol{g}_{c,r}}{\boldsymbol{K}_{c,r}\hat{\boldsymbol{f}}_{c}^{(\text{old})}}\right).  $$

#### Implementation

**Projection and rotation matrices** Projection matrices ***P***^*ℓ*^ were implemented such that they also model collimator-detector response as a distance-dependent Gaussian function [27]. Rotation matrices ***R***^*ℓ*^ were implemented using Gaussian interpolation [28]. Acquisition times $\tau _{c,r}^{\ell }$ were calculated during list-mode data processing.

**Transformation matrices** To estimate the motion between respiratory windows *r* and *r*^′^, a sequence of respiratory-gated images was reconstructed based on data vectors $\sum _{c=1}^{C}\boldsymbol {g}_{c,r}$ using the standard MLEM algorithm. We ran the algorithm for 15 iterations and applied post-reconstruction Gaussian smoothing with a standard deviation of 1 voxel. We chose *r*^′^=0.5*R* as the reference respiratory window from which the myocardium-containing region of interest (ROI) was segmented using an approach similar to the one introduced by Germano et al. [29]. Voxels outside the ROI were set to zero, and the other respiratory windows were registered with the reference window by assuming rigid-body translation and using the sum of squared differences as the term to be minimized with Gauss-Newton optimization [30]. The motivation for segmenting myocardium from the reference window was to focus the image registration on the myocardial region because respiratory motion of the heart may be different than that of extracardiac structures [22]. After computing the translation vectors, their directions were reversed to finally form the matrices $\phantom {\dot {i}\!}\boldsymbol {M}_{r'\rightarrow r}$ applying linear interpolation.

**Reconstruction details** In both phantom and patient studies, the reconstructions with Eqs. (2) and (6) were accelerated by using the ordered subsets variant of the EM algorithm (OSEM) [31]. The data were arranged to 8 subsets, and the algorithm was run for 10 iterations. In the phantom experiment, the stationary reference data were reconstructed with standard OSEM algorithm, and the moving data were reconstructed with OSEM, RBM-OSEM, and JMC-OSEM algorithms. The computation times for each method were recorded. In the patient studies, the data were reconstructed with OSEM, RBM-OSEM, and JMC-OSEM algorithms.

In patient studies, the reconstructed transaxial images were manually rotated to short-axis images using Gaussian interpolation. Extracardiac activity was masked, and three-dimensional post-reconstruction Gaussian filtering with full width at half maximum of 13.3 mm was applied. In phantom experiment, no rotation was necessary as the coronal plane was parallel to the short-axis plane.

### Image analysis: phantom experiment

**Fig. 1:**
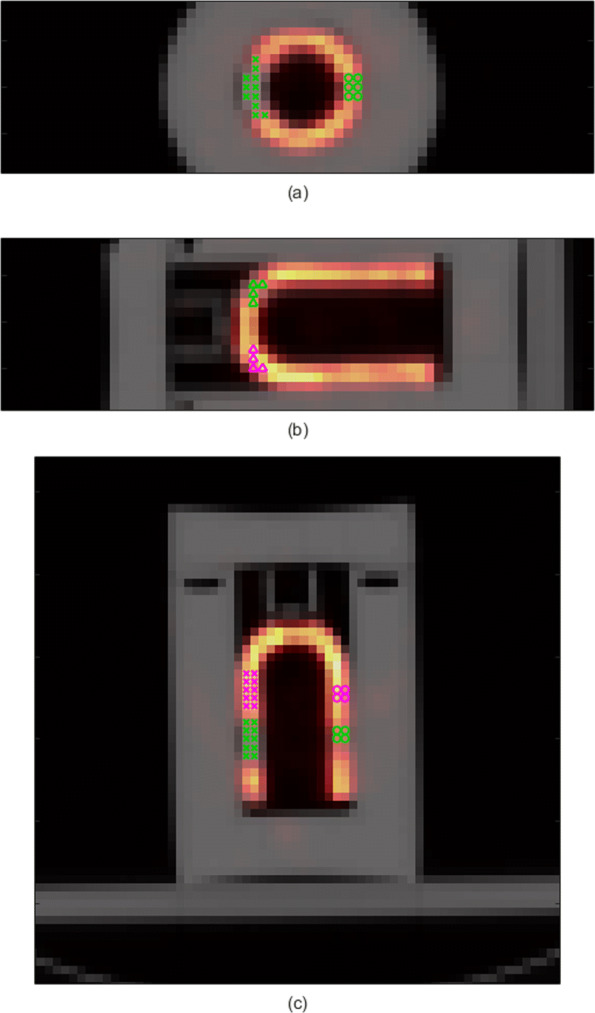
Defect and normal myocardial regions. Stationary SPECT image fused with CT image is displayed. Coronal (**a**), sagittal (**b**), and transaxial (**c**) views are displayed. The defect regions are displayed with green markers and the normal myocardial regions (references) with purple markers. The large segmental defect is displayed with crosses (×; in **a** and **c**) and the two smaller, cubical defects are displayed with circles (*o*; in **a** and **c**) and triangles (*Δ*; in **b**)

In order to quantitatively study the visibility of cardiac defects, we computed the contrast between each defect region and a corresponding normal myocardial region according to the study of Kortelainen et al. [18]. Briefly, the CT image of the stationary phantom was converted to *μ*-map using piecewise linear conversion [32]. This *μ*-map was resampled to SPECT matrix size and coregistered with SPECT image applying normalized gradient fields method [33]. This *μ*-map was then converted back to Hounsfield units (HUs). This was done to identify the defect regions, which had higher HU values than the surrounding water. An HU value of 50 was used as the threshold to binarize the converted *μ*-map, which permitted the selection of defect-containing regions. The normal myocardial regions (references) were determined as regions of similar shape and size, located adjacent to the defect regions (Fig. 1).

Having computed the coordinates of defect and reference regions, OSEM, RBM-OSEM, and JMC-OSEM reconstructed moving images were registered with the stationary image and post-reconstruction Gaussian filtering with a standard deviation of 1.0 was applied. For each image, contrast for each defect was computed as [18]

7 $$  \text{Contrast} = \frac{A_{\text{reference}} - A_{\text{defect}}}{A_{\text{reference}}},  $$

where *A*_reference_ and *A*_defect_ are average voxel values on reference ROI and defect ROI, respectively. In addition, mean squared error (MSE) of each of the moving images was computed, considering the stationary image as reference.

### Image analysis: patient studies

Based on the results from the phantom experiment, we opted to analyze only the OSEM and JMC-OSEM reconstructed patient images, excluding the RBM-OSEM images. The analysis was performed with Quantitative Gated SPECT (QGS) 2012 program (Cedars-Sinai Medical Center, USA) [34]. Studied parameters included LV end-diastolic volume (EDV), end-systolic volume (ESV), stroke volume (SV), ejection fraction (EF), and wall motion (WM) and wall thickening (WT) assessed in anterior, lateral, inferior, septal, and apical wall segments. In addition, we computed global wall motion (WM _GLB_) and global wall thickening (WT _GLB_) as average values over these five segments.

In phase analysis, QGS fits a first Fourier harmonic curve to the time-activity data of each myocardial sampling point and records amplitude and phase of each curve [35]. Before progressing, the program discards 5% of the phase angles whose corresponding amplitude values are the lowest. The remaining phase angles are binned into a histogram using 360 bins, and histogram bandwidth (BW), standard deviation (StD), and entropy (ENT) are computed to describe the phase angle distribution.

In order to evaluate the effect of motion compensation on myocardial perfusion, we applied the “Freeze” command of QGS. This command warps the myocardial activity from all cardiac bins *c* to the end-diastolic bin through landmark-based image registration and sums them together, thus eliminating cardiac contractile motion [36]. Perfusion of these “motion-frozen” images was assessed using the same five-segment polar map as in the assessment of wall motion and wall thickening.

Statistical significance of differences between motion-compensated and non-motion-compensated data was evaluated with SPSS Statistics 23 (IBM Corporation, USA). If the data were normally distributed based on the Shapiro-Wilk test (*p* > 0.05), we used paired-samples *t* test for comparisons. If the data were not normally distributed (*p* < 0.05), we used Wilcoxon signed-rank test for comparisons.

## Results

### Phantom experiment

**Fig. 2:**
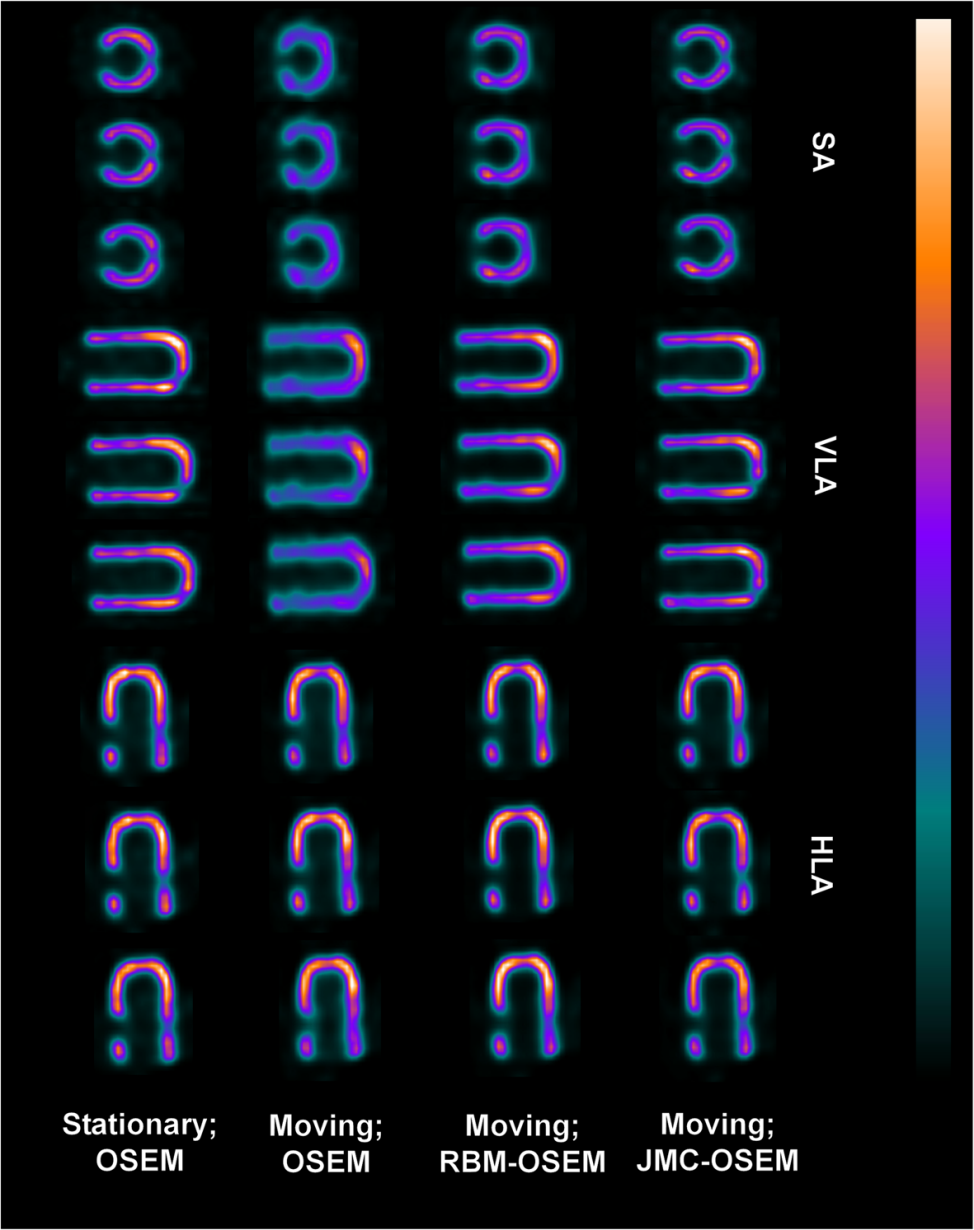
Three consecutive short axis (SA), vertical long axis (VLA), and horizontal long axis (HLA) views of the reconstructed phantom images. The first column displays the stationary phantom reconstructed with OSEM, the second column displays the moving phantom reconstructed with OSEM, the third column displays the moving phantom reconstructed with RBM-OSEM, and the fourth column displays the moving phantom reconstructed with JMC-OSEM. Corresponding polar maps are presented in Fig. 3

**Fig. 3:**
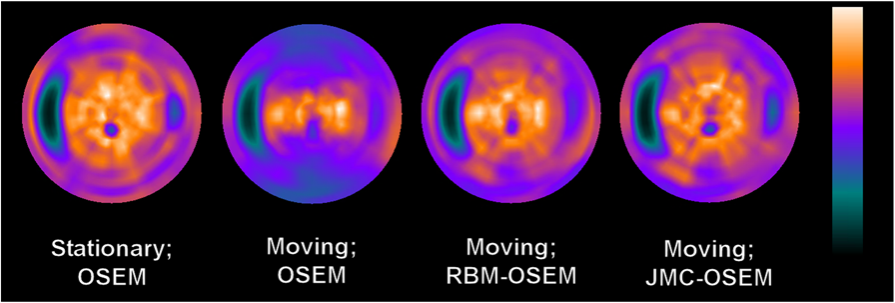
Polar maps of the reconstructed phantom images

The computation times for each method are given in Table 1. Figures 2 and 3 present images of the phantom as stationary and reconstructed with OSEM, moving and reconstructed with OSEM, moving and reconstructed with RBM-OSEM, and moving and reconstructed with JMC-OSEM. Contrasts for individual defects and the MSE values of images are given in Table 2. While RBM-OSEM successfully reduced respiratory blur compared to the standard OSEM as seen from the lower MSE value, it could only partially recover the visibility of cardiac defects. The visibility of defects was further improved with JMC-OSEM.

**Table 1:** Computation times of methods used in phantom experiment

Method	Time (s)
OSEM	1466.46
RBM-OSEM	2645.79
JMC-OSEM	18,351.60

These reconstructions were performed on Dell Precision T7400 (Intel(R) Xeon(R) CPU E5410 @ 2.33 GHz and 32 GB DDR2 FB-DIMM)

**Table 2:** Results from phantom image analysis

	Contrast			MSE (·10^−5^)
	Segment	Cube _lateral_	Cube _apical_	
OSEM (static)	0.6307	0.3209	0.3810	0
OSEM (moving)	0.6050	0.2504	0.3069	8.5406
RBM-OSEM	0.6342	0.2869	0.3053	2.7190
JMC-OSEM	0.6340	0.3423	0.3541	2.0795

### Patient studies

Respiratory motion magnitude observed in each patient study was computed as Euclidean distance between respiratory windows 1 and 7. For this study population, the (mean ± standard deviation) respiratory motion magnitude was 10.9 ± 4.9 mm.

**Fig. 4:**
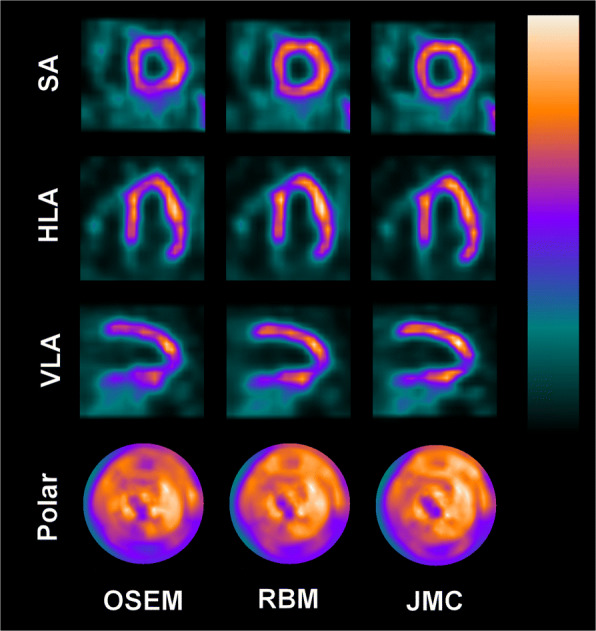
“Motion-frozen” short axis (SA), horizontal long axis (HLA), and vertical long axis (VLA) views and polar maps of the reconstructed patient images as displayed by QGS software. Post-filtering has been omitted to better visualize the differences caused by motion compensation. For this patient, measured respiratory motion magnitude was 13.3 mm

**Fig. 5:**
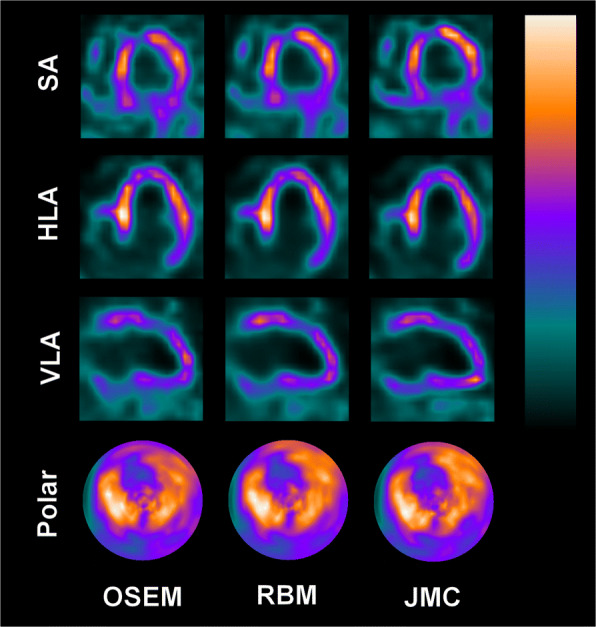
“Motion-frozen” short axis (SA), horizontal long axis (HLA), and vertical long axis (VLA) views and polar maps of the reconstructed patient images as displayed by QGS software. Post-filtering has been omitted to better visualize the differences caused by motion compensation. For this patient, measured respiratory motion magnitude was 12.3 mm

**Fig. 6:**
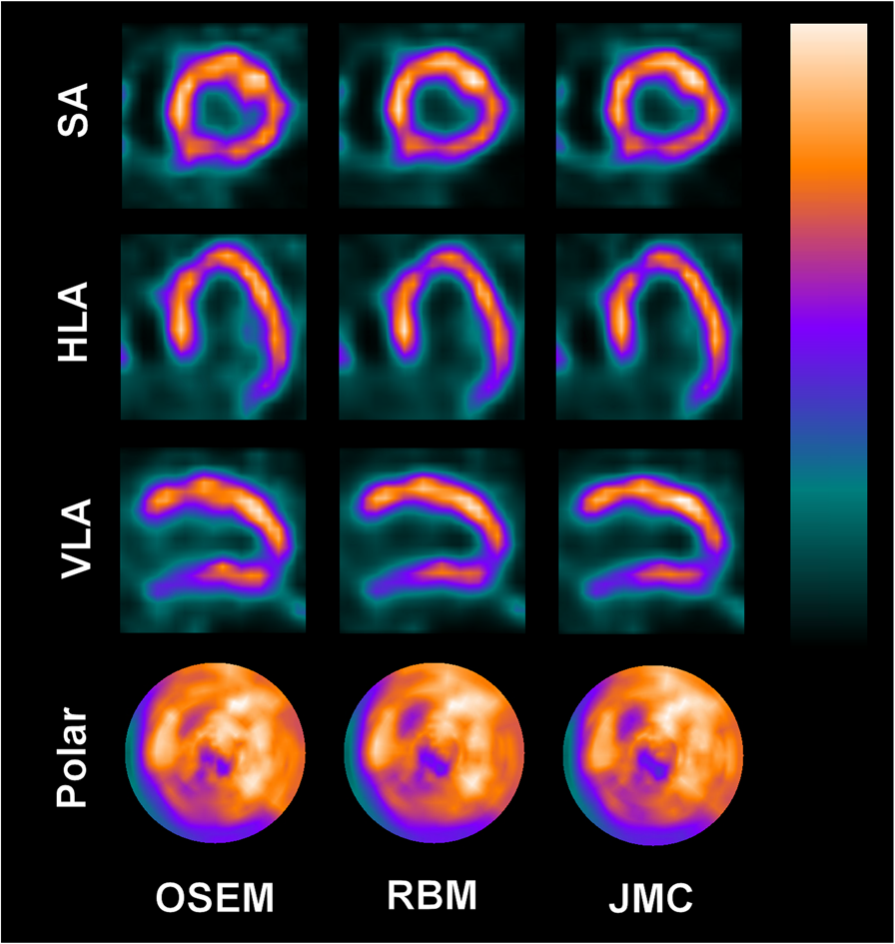
“Motion-frozen” short axis (SA), horizontal long axis (HLA), and vertical long axis (VLA) views and polar maps of the reconstructed patient images as displayed by QGS software. Post-filtering has been omitted to better visualize the differences caused by motion compensation. For this patient, measured respiratory motion magnitude was 15.3 mm

Figures 4, 5, and 6 present “motion-frozen” images of three patient cases, with the images reconstructed with OSEM, RBM-OSEM, and JMC-OSEM algorithms. Motion compensation either retained or improved image quality of the “motion-frozen” images, depending on the measured respiratory motion magnitude. The improvements could be seen as higher contrast between the myocardium and LV cavity and better visibility of hypoperfused regions. The best visibility of hypoperfused regions was achieved with JMC-OSEM.

All QGS analysis results are given in Table 3. Use of JMC increased EDV and ESV and decreased EF compared to standard reconstructions (*p* < 0.001) and left SV unaffected (*p* > 0.05). Use of JMC also decreased wall motion in inferior (*p* < 0.05) and lateral (*p* < 0.01) walls, and also globally (*p* < 0.05). In addition, it decreased wall thickening in septal and inferior walls (*p* < 0.05). Use of JMC increased phase analysis parameter StD (*p* < 0.05). Use of JMC also decreased myocardial perfusion in lateral and septal walls (*p* < 0.01) and in apex (*p* < 0.05), but left anterior and inferior walls unaffected (*p* > 0.05).

**Table 3:** Results from QGS analysis

Parameter	OSEM	JMC-OSEM	*p*
EDV (mL)	94.6 ± 28.5	97.5 ± 29.3	< 0.001
ESV (mL)	40.9 ± 21.7	43.3 ± 22.7	< 0.001*
SV (mL)	53.5 ± 11.7	54.2 ± 11.5	0.058*
EF (%)	58.6 ± 9.9	57.7 ± 9.6	< 0.001
WM_ANT_ (mm)	8.38 ± 1.18	8.37 ± 1.29	0.774
WM_LAT_ (mm)	8.84 ± 1.32	8.72 ± 1.31	0.008
WM_INF_ (mm)	5.93 ± 1.50	5.80 ± 1.45	0.034
WM_SEP_ (mm)	4.44 ± 1.73	4.37 ± 1.70	0.198
WM_APX_ (mm)	6.11 ± 2.12	6.00 ± 2.05	0.121
WM_GLB_ (mm)	6.74 ± 1.26	6.65 ± 1.24	0.023
WT_ANT_ (%)	32.5 ± 7.6	32.5 ± 7.5	0.909
WT_LAT_ (%)	32.2 ± 7.6	31.6 ± 7.5	0.061
WT_INF_ (%)	28.4 ± 7.5	27.8 ± 7.3	0.019
WT_SEP_ (%)	33.8 ± 8.2	33.1 ± 8.1	0.049
WT_APX_ (%)	50.8 ± 15.2	50.3 ± 15.3	0.171
WT_GLB_ (%)	35.6 ± 8.4	35.1 ± 8.3	0.066
BW (^∘^)	60.6 ± 29.3	65.5 ± 31.0	0.066
StD (^∘^)	18.4 ± 9.3	19.9 ± 8.5	0.024
ENT (%)	61.1 ± 5.6	61.4 ± 4.7	0.400
PERF_ANT_ (%)	72.3 ± 6.1	73.2 ± 5.8	0.094
PERF_LAT_ (%)	74.8 ± 4.9	73.6 ± 4.9	0.004
PERF_INF_ (%)	62.4 ± 8.3	62.7 ± 8.6	0.481
PERF_SEP_ (%)	69.8 ± 5.8	68.5 ± 6.0	0.003
PERF_APX_ (%)	73.9 ± 9.4	72.6 ± 9.4	0.010*

Results are expressed as mean ± standard deviation

Asterisk (*) denotes use of Wilcoxon signed-rank test instead of paired-samples *t* test

*EDV* end-diastolic volume, *ESV* end-systolic volume, *SV* stroke volume, *EF* ejection fraction, *WM* wall motion, *WT* wall thickening, *BW* bandwidth, *StD* standard deviation, *ENT* entropy, *PERF* perfusion, *ANT* anterior, *LAT* lateral, *INF* inferior, *SEP* septal, *APX* apical, *GLB* global

## Discussion

In this work, we studied the performance of two respiratory motion compensation strategies. Both methods, RBM-OSEM and JMC-OSEM, reduced respiratory motion blur and improved visibility of hypoperfused regions compared to the standard OSEM algorithm; however, JMC-OSEM proved to be superior to RBM-OSEM. Thus, we chose the JMC-OSEM algorithm to study the effect of respiratory motion compensation on quantification of LV functional parameters.

These motion compensation algorithms are just a few of several methods that have been presented over the years, and they were chosen for the present work because they fit well in the MLEM framework which is currently the recommended way to reconstruct myocardial perfusion images [37]. Another appropriate method is the popular reconstruct-transform-average (RTA) algorithm [12]. The reason why RTA was not included in this study was because we concluded earlier using phantom data that, in terms of MSE, RBM-OSEM outperformed it in case where the patient’s breathing pattern included a baseline shift (Additional file 1: Supplementary material).

It was not obvious for us whether RBM-OSEM could outperform JMC-OSEM, so we included both algorithms in this study. While JMC-OSEM was superior to RBM-OSEM in terms of image quality (MSE), one must decide whether it is worth the computational cost; as one can observe from Eq. (6), each iteration of JMC requires *R* forward projections and 2*R* backprojections, whereas RBM requires just 1 forward projection and 2 backprojections, as shown in Eq. (2). Appropriately, in this work, reconstruction of phantom data of 10 respiratory windows with JMC-OSEM lasted nearly 7 times longer than reconstruction with RBM-OSEM (Table 1). This apparent discrepancy of 7 times longer computation time instead of 10 is related to multiplication with sparse matrices $\phantom {\dot {i}\!}\boldsymbol {M}_{r'\rightarrow r}$ in JMC-OSEM and ***B***_*c*_ in RBM-OSEM. ***B***_*c*_ is a weighted average of *R* different $\phantom {\dot {i}\!}\boldsymbol {M}_{r'\rightarrow r}$ matrices and thus contains more non-zero elements than a single $\phantom {\dot {i}\!}\boldsymbol {M}_{r'\rightarrow r}$ matrix, which increases the computation time of RBM-OSEM algorithm.

Respiratory motion has been demonstrated to decrease the apparent myocardial activity especially in the anterior and inferior walls in several studies [11, 18, 38, 39], and the same observation was made in the present phantom experiment (Figs. 2 and 3). However, we did not obtain similar results from the patient studies (Table 3)—some JMC reconstructions displayed higher perfusion values in anterior and inferior walls, but overall, the differences were statistically non-significant. One explanation could be the way QGS presents the perfusion results: they are scaled to the maximal myocardial count value. Unfortunately, we cannot obtain absolute “motion-frozen” perfusion values for comparisons from QGS. On the other hand, the observed perfusion decreases in lateral, septal, and apical walls are concordant with the results of Ko et al. [39].

Use of JMC increased LV EDV and ESV, as could be expected based on previous studies [17, 39, 40]. In addition, the decrease of global wall motion as a result of JMC is concordant with the simulation results of Bitarafan-Rajabi et al. [40], although one could have expected a larger decrease. Although the change was statistically non-significant, we observed a small decrease in global wall thickening, which was also suggested by Bitarafan-Rajabi et al. [40].

Interestingly, we found that JMC caused a small but statistically significant increase in phase analysis parameter StD. This finding indicates that respiratory motion is a mechanism that partially conceals mechanical dyssynchrony. As far as we know, this finding has not been reported before. In the previous study by Kortelainen et al., no differences in phase analysis parameters were found between respiratory-gated and non-respiratory-gated images [17]. This might be due to the fact that the authors used only 15-s-long time window in projection image binning, as opposed to the present study where we used all the acquired emission photons that occurred during accepted R-R intervals (∼ 30 s). Therefore, the effect of their respiratory motion compensation method may have been overshadowed by the effect of increased noise.

This study had some limitations. First, the number of patients was low. Unfortunately, the camera used in the current study was upgraded to a new model which prevented acquisition of a larger study population. Second, in our current method, we estimate the respiratory motion before the final reconstruction by pre-reconstructing the respiratory windows. However, it was demonstrated by Song et al. that simultaneous reconstruction and motion estimation could be more effective in compensation of respiratory motion [41]. Third, besides the phantom experiment, we are unable to provide a reference “ground truth” data for the patient studies. However, as pointed out in the previous paragraphs, a part of our results are concordant with the ones obtained in previous studies, which enhances the credibility of our work. Fourth, the applied motion model (translation only) is simplistic to accurately describe real cardiac respiratory motion. However, since the pre-reconstructions of respiratory windows are likely to suffer from limited-angle errors due to irregular breathing [22], we opted for the most robust motion model (only three parameters to be estimated).

The degree of visual improvement obtained using respiratory motion compensation likely depends on the respiratory motion magnitude. Previously, Pretorius et al. established linear regression models for segmental count differences as a function of respiratory motion in a large study population (*n* = 1103) [42]. The larger the respiratory motion was, the larger were the count differences between respiratory motion-compensated and non-compensated reconstructions [42]. Although our study population is too small to definitely establish such trends, our results agree with those of Pretorius et al. For patients whose respiratory motion magnitude was smaller than 5 mm, the visual changes due to motion compensation were virtually non-existent. For the three patients presented in Figs. 4, 5, and 6, use of RBM and JMC improved the visibility of hypoperfused myocardial regions and the contrast between myocardium and LV cavity. These promising results warrant further studies and suggest that respiratory motion compensation could be performed routinely in near future.

## Conclusions

We studied two respiratory motion compensation expectation maximization algorithms, RBM-OSEM and JMC-OSEM, in the context of myocardial perfusion SPECT imaging. The algorithms were validated with a phantom experiment. The JMC-OSEM algorithm was found to be superior in terms of image quality and was thus subsequently chosen to reconstruct clinical cardiac-gated myocardial perfusion images. Use of motion compensation slightly increased phase analysis-quantified mechanical dyssynchrony. It also improved image quality and had minor effects on left ventricular volumes, wall motion, and wall thickening.

## Supplementary Information

**Additional file 1** Effect of data conserving respiratory motion compensation on left ventricular functional parameters assessed in gated myocardial perfusion SPECT: Supplement.;

## Data Availability

Data will not be shared because they will be used in the ongoing PhD project.

## References

[1] Klocke FJ, Baird MG, Bateman TM, Berman DS, Carabello BA, Cerqueira MD, DeMaria AN, Kennedy JW, Lorell BH, Messer JV, O’Gara PT, Russell RO, St. John Sutton MG, Udelson JE, Verani MS, Williams KA (2003). ACC/AHA/ASNC guidelines for the clinical use of cardiac radionuclide imaging—executive summary: a report of the American College of Cardiology/American Heart Association task force on practice guidelines (ACC/AHA/ASNC committee to revise the 1995 guidelines for the clinical use of radionuclide imaging). Circulation.

[2] Germano G, Erel J, Lewin H, Kavanagh PB, Berman DS (1997). Automatic quantitation of regional myocardial wall motion and thickening from gated technetium-99m sestamibi myocardial perfusion single-photon emission computed tomography. J Am Coll Cardiol.

[3] Neill J, Harbinson M, Adgey J (2010). Myocardial wall motion and thickening assessment in early gated SPECT images of acute coronary syndrome patients likely to have inferolateral perfusion defects. Int J Cardiovasc Imaging.

[4] Karimi-Ashtiani S, Arsanjani R, Fish M, Kavanagh P, Germano G, Berman D, Slomka P (2012). Direct quantification of left ventricular motion and thickening changes using rest-stress myocardial perfusion SPECT. J Nucl Med.

[5] Lairez O, Cognet T, Dercle L, Méjean S, Berry M, Bastié D, Richaud R, Gautier M, Fouilloux A, Galinier M, Carrié D, Massabuau P, Berry I (2014). Prediction of all-cause mortality from gated-SPECT global myocardial wall thickening: comparison with ejection fraction and global longitudinal 2D-strain. J Nucl Cardiol.

[6] Yang W, Zhang F, Tang H, Shao X, Wang J, Wang X, Shao X, Xin W, Yang L, Zhou W, Wang Y (2018). Summed thickening score by myocardial perfusion imaging: a risk factor of left ventricular remodeling in patients with myocardial infarction. J Nucl Cardiol.

[7] Chen J, Garcia EV, Folks RD, Cooke CD, Faber TL, Tauxe EL, Iskandrian AE (2005). Onset of left ventricular mechanical contraction as determined by phase analysis of ECG-gated myocardial perfusion SPECT imaging: development of a diagnostic tool for assessment of cardiac mechanical dyssynchrony. J Nucl Cardiol.

[8] Henneman MM, Chen J, Dibbets-Schneider P, Stokkel MP, Bleeker GB, Ypenburg C, van der Wall EE, Schalij MJ, Garcia EV, Bax JJ (2007). Can LV dyssynchrony as assessed with phase analysis on gated myocardial perfusion SPECT predict response to CRT?. J Nucl Med.

[9] Boogers MM, Van Kriekinge SD, Henneman MM, Ypenburg C, Bommel RJV, Boersma E, Dibbets-Schneider P, Stokkel MP, Schalij MJ, Berman DS, Germano G, Bax JJ (2009). Quantitative gated SPECT-derived phase analysis on gated myocardial perfusion SPECT detects left ventricular dyssynchrony and predicts response to cardiac resynchronization therapy. J Nucl Med.

[10] Wang Y, Riederer SJ, Ehman RL (1995). Respiratory motion of the heart: kinematics and the implications for the spatial resolution in coronary imaging. Magn Reson Med.

[11] Kovalski G, Israel O, Keidar Z, Frenkel A, Sachs J, Azhari H (2007). Correction of heart motion due to respiration in clinical myocardial perfusion SPECT scans using respiratory gating. J Nucl Med.

[12] Polycarpou I, Tsoumpas C, Marsden PK (2012). Analysis and comparison of two methods for motion correction in PET imaging. Med Phys.

[13] Qi W, Yang Y, Wernick MN, Pretorius PH, King MA (2015). Compensation of acquisition variations in respiratory-gated SPECT with joint statistical reconstruction. 2015 IEEE 12th International Symposium on Biomedical Imaging (ISBI).

[14] Polycarpou I, Chrysanthou-Baustert I, Demetriadou O, Parpottas Y, Panagidis C, Marsden PK, Livieratos L (2017). Impact of respiratory motion correction on SPECT myocardial perfusion imaging using a mechanically moving phantom assembly with variable cardiac defects. J Nucl Cardiol.

[15] Qi W, Yang Y, Song C, Wernick MN, Pretorius PH, King MA (2017). 4-D reconstruction with respiratory correction for gated myocardial perfusion SPECT. IEEE Trans Med Imaging.

[16] Buechel RR, Husmann L, Pazhenkottil AP, Nkoulou R, Herzog BA, Burger IA, Ghadri JR, Wolfrum M, Kaufmann PA (2010). Myocardial perfusion imaging with real-time respiratory triggering: impact of inspiration breath-hold on left ventricular functional parameters. J Nucl Cardiol.

[17] Kortelainen MJ, Koivumäki TM, Vauhkonen MJ, Hedman MK, Kärkkäinen ST, Niño Quintero J, Hakulinen MA. Respiratory motion reduction with a dual gating approach in myocardial perfusion SPECT: effect on left ventricular functional parameters. J Nucl Cardiol. 2017;1–9. 10.1007/s12350-017-0844-9. 10.1007/s12350-017-0844-9 28303474

[18] Kortelainen MJ, Koivumäki TM, Vauhkonen MJ, Hakulinen MA (2019). Effect of respiratory motion on cardiac defect contrast in myocardial perfusion SPECT: a physical phantom study. Ann Nucl Med.

[19] Sakaguchi K, Hosono M, Otsuka M, Hanaoka K, Usami K, Uto T, Ishii K (2013). Dynamic sequence respiratory gated perfusion pulmonary SPECT without external tracking device. Ann Nucl Med.

[20] Pan J, Tompkins WJ (1985). A real-time QRS detection algorithm. IEEE Trans Biomed Eng.

[21] Bacharach SL, Bonow RO, Green MV (1990). Comparison of fixed and variable temporal resolution methods for creating gated cardiac blood-pool image sequences. J Nucl Med.

[22] Dey J, Segars WP, Pretorius PH, Walvick RP, Bruyant PP, Dahlberg S, King MA (2010). Estimation and correction of cardiac respiratory motion in SPECT in the presence of limited-angle effects due to irregular respiration. Med Phys.

[23] Kortelainen MJ, Koivumäki TM, Vauhkonen MJ, Hakulinen MA (2019). Time-modified OSEM algorithm for more robust assessment of left ventricular dyssynchrony with phase analysis in ECG-gated myocardial perfusion SPECT. EJNMMI Phys.

[24] Lange K, Carson R (1984). EM reconstruction algorithms for emission and transmission tomography. J Comput Assist Tomogr.

[25] Reyes M, Malandain G, Koulibaly PM, González-Ballester MA, Darcourt J (2007). Model-based respiratory motion compensation for emission tomography image reconstruction. Phys Med Biol.

[26] Rahmim A, Cheng J-C, Dinelle K, Shilov M, Segars WP, Rousset OG, Tsui BMW, Wong DF, Sossi V (2008). System matrix modelling of externally tracked motion. Nucl Med Commun.

[27] Anger HO (1964). Scintillation camera with multichannel collimators. J Nucl Med.

[28] Wallis JW, Miller TR (1997). An optimal rotator for iterative reconstruction. IEEE Trans Med Imaging.

[29] Germano G, Kavanagh PB, Su H-T, Mazzanti M, Kiat H, Hachamovitch R, Van Train KF, Areeda JS, Berman DS (1995). Automatic reorientation of three-dimensional, transaxial myocardial perfusion SPECT images. J Nucl Med.

[30] Ashburner J, Friston K (2007). Rigid body registration, 1st edn. Statistical Parametric Mapping: The Analysis of Functional Brain Images.

[31] Hudson HM, Larkin RS (1994). Accelerated image reconstruction using ordered subsets of projection data. IEEE Trans Med Imaging.

[32] Bai C, Shao L, Da Silva AJ, Zhao Z (2003). A generalized model for the conversion from CT numbers to linear attenuation coefficients. IEEE Trans Nucl Sci.

[33] Modersitzki J (2009). Normalized gradient fields. FAIR: Flexible Algorithms for Image Registration.

[34] Germano G, Kavanagh PB, Slomka PJ, Van Kriekinge SD, Pollard G, Berman DS (2007). Quantitation in gated perfusion SPECT imaging: the Cedars-Sinai approach. J Nucl Cardiol.

[35] Van Kriekinge SD, Nishina H, Ohba M, Berman DS, Germano G (2008). Automatic global and regional phase analysis from gated myocardial perfusion SPECT imaging: application to the characterization of ventricular contraction in patients with left bundle branch block. J Nucl Med.

[36] Slomka PJ, Nishina H, Berman DS, Kang X, Akincioglu C, Friedman JD, Hayes SW, Aladl UE, Germano G (2004). “Motion-frozen” display and quantification of myocardial perfusion. J Nucl Med.

[37] Verberne HJ, Acampa W, Anagnostopoulos C, Ballinger J, Bengel F, De Bondt P, Buechel RR, Cuocolo A, van Eck-Smit BLF, Flotats A, Hacker M, Hindorf C, Kaufmann PA, Lindner O, Ljungberg M, Lonsdale M, Manrique A, Minarik D, Scholte AJHA, Slart RHJA, Trägårdh E, de Wit TC, Hesse B (2015). EANM procedural guidelines for radionuclide myocardial perfusion imaging with SPECT and SPECT/CT: 2015 revision. Eur J Nucl Med Mol Imaging.

[38] Segars WP, Tsui BM (2002). Study of the efficacy of respiratory gating in myocardial SPECT using the new 4-D NCAT phantom. IEEE Trans Nucl Sci.

[39] Ko C-L, Wu Y-W, Cheng M-F, Yen R-F, Wu W-C, Tzen K-Y (2015). Data-driven respiratory motion tracking and compensation in CZT cameras: a comprehensive analysis of phantom and human images. J Nucl Cardiol.

[40] Bitarafan-Rajabi A, Rajabi H, Rastgou F, Sharafi AA (2009). Effect of respiratory motion on quantitative myocardial gated SPECT: a simulation study. Ann Nucl Med.

[41] Song C, Yang Y, Wernick MN, Pretorius PH, King MA (2016). Optimizing motion correction in reconstruction of respiratory-gated SPECT. 2016 IEEE 13th International Symposium on Biomedical Imaging (ISBI).

[42] Pretorius PH, Johnson KL, Dahlberg ST, King MA. Investigation of the physical effects of respiratory motion compensation in a large population of patients undergoing Tc-99m cardiac perfusion SPECT/CT stress imaging. Nucl Cardiol, J. 2017;1–6. 10.1007/s12350-017-0890-3. 10.1007/s12350-017-0890-3 PMC7714447 28432671

