# Supplementary material for: Effect of data conserving respiratory motion compensation on left ventricular functional parameters assessed in gated myocardial perfusion SPECT
Source: EJNMMI Phys. 2021 Jan 21;8:7. doi: 10.1186/s40658-021-00355-w (PMC7818343; doi:10.1186/s40658-021-00355-w)
Supplement: Supplementary file 1 — Additional file 1 Effect of data conserving respiratory motion compensation on left ventricular functional parameters assessed in gated myocardial perfusion SPECT: Supplement.; [file 40658_2021_355_MOESM1_ESM.pdf]

SUPPLEMENT

Effect of data conserving respiratory motion compensation on left ventricular functional parameters assessed in gated myocardial perfusion SPECT: Supplement

Matti J Kortelainen[1,2*], Tuomas M Koivumäki[3], Marko J Vauhkonen[1] and Mikko A Hakulinen[1,2]

*Correspondence:
matti.kortelainen@uef.fi
[1]Department of Applied Physics, University of Eastern Finland, POB 1627, FI-70211 Kuopio, Finland
Full list of author information is available at the end of the article

Background

Reconstruct-transform-average (RTA) is a relatively straightforward method to perform respiratory motion compensation. In RTA, individual respiratory windows are reconstructed with, for example, standard OSEM algorithm, these reconstructed images are coregistered, and their average voxel values are computed [1]. However, when respiratory gating is performed using amplitude windows and the patient's breathing pattern is not regular, there is a chance that certain respiratory windows receive very few or no counts at all at some projection angles. Reconstructing this kind of data may lead to so-called limited-angle artifacts [2], which may hamper the final RTA reconstruction. In this supplementary work, we compare the performance of RTA method to the respiratory blur modeling (RBM) method in a situation where the patient's breathing pattern contains a drastic baseline shift.

Methods

The phantom and its imaging procedures used in this supplementary work were the same as the ones used in the main article. However, for the moving phantom data, all acquired counts (30 seconds) were used in the binning of respiratory-gated projections. Then, for respiratory windows 1–5, projection images 17–32 and 49–64 were set to zero, and for respiratory windows 6–10, projection images 1–16 and 33-48 were set to zero. This was done to simulate a worst-case situation where the patient's breathing pattern changes in the middle of the image acquisition: first the patient breathes so that the expiratory reserve volume in the lungs is low, then the patient inhales deeply and continues tidal breathing with higher expiratory reserve volume. For reference, the stationary phantom data were binned such that the data occurring during the first 15 seconds at each projection angle were used.

The stationary phantom data were reconstructed using OSEM algorithm with 10 iterations and 8 subsets. Moving phantom data were reconstructed with OSEM, RTA-OSEM and RBM-OSEM algorithms. In order to compare only the effects of different reconstruction algorithms, we used the same motion matrices as in the main article, in order to not introduce errors in image registration due to limited-angle artifacts.

The reconstructed images were analyzed in the same way as in the main article. The SPECT images were registered with the stationary CT image from which the

defect-containing regions of interest (ROIs) and reference ROIs were determined, and the contrasts between defects and their corresponding reference regions were computed. In addition, mean-squared errors (MSEs) were computed, considering the stationary image as the reference.

Results and Discussion

The reconstructed images are presented in Figure 1. The contrast and MSE results are presented in Table 1. While RTA-OSEM provided even higher contrast values for defects than RBM-OSEM, it had over 8 times higher MSE value than RBM-OSEM. This is the result of limited-angle artifacts in individually reconstructed respiratory windows; averaging images does not make these artifacts disappear. This is also visible for human eye in Figure 1: the RTA-OSEM reconstruction looks like as if the activity has "spread" from the myocardial walls to the ventricular cavity. RBM-OSEM, on the other hand, does not suffer from these artifacts.

While the breathing pattern implemented in this supplementary work may never occur in clinical circumstances, it proves an important point about RTA-OSEM: it is not immune to limited-angle artifacts.

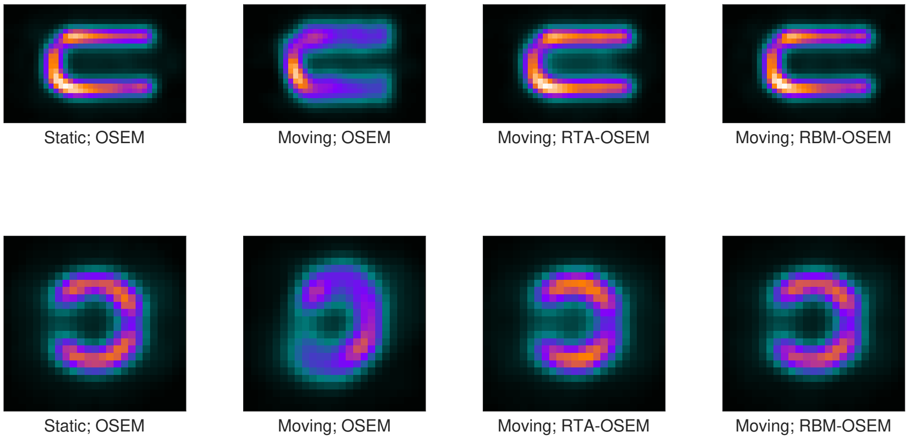

Figure 1 Sagittal (top row) and coronal (bottom row) views of the reconstructed phantom images. The first column displays the stationary phantom reconstructed with OSEM, the second column displays the moving phantom reconstructed with OSEM, the third column displays the moving phantom reconstructed with RTA-OSEM and the fourth column displays the moving phantom reconstructed with RBM-OSEM.

Conclusions

We have shown in this supplementary work that RTA-OSEM, while perhaps straightforward to implement, is susceptible to limited-angle artifacts when the patient's breathing pattern includes baseline shifts. As a result, it produces images that are, in terms of MSE, less similar to ground truth images than images reconstructed with RBM-OSEM. No limited-angle artifacts could be observed in RBM-OSEM images.

Author details
[1]Department of Applied Physics, University of Eastern Finland, POB 1627, FI-70211 Kuopio, Finland. [2]Diagnostic Imaging Center, Kuopio University Hospital, Kuopio, Finland. [3]Department of Medical Physics, Central Finland Central Hospital, Jyväskylä, Finland.

References

1. Polycarpou, I., Tsoumpas, C., Marsden, P.K.: Analysis and comparison of two methods for motion correction in PET imaging. Med Phys **39**(10), 6474–6483 (2012)
2. Dey, J., Segars, W.P., Pretorius, P.H., Walvick, R.P., Bruyant, P.P., Dahlberg, S., King, M.A.: Estimation and correction of cardiac respiratory motion in SPECT in the presence of limited-angle effects due to irregular respiration. Med Phys **37**(12), 6453–6465 (2010)

Tables

Table 1 Results from phantom image analysis

	Contrast			MSE
	Segment	$\text{Cube}_{\text{lateral}}$	$\text{Cube}_{\text{apical}}$	$(\cdot 10^{-5})$
OSEM (static)	0.6097	0.3598	0.3572	0
OSEM (moving)	0.5854	0.2709	0.3211	19.8006
RTA-OSEM	0.6260	0.3857	0.3498	3.6359
RBM-OSEM	0.6271	0.3533	0.3467	0.4384
